# Improving the Heading Accuracy in Indoor Pedestrian Navigation Based on a Decision Tree and Kalman Filter

**DOI:** 10.3390/s20061578

**Published:** 2020-03-12

**Authors:** Guanghui Hu, Weizhi Zhang, Hong Wan, Xinxin Li

**Affiliations:** 1 State Key Laboratory of Transducer Technology, Shanghai Institute of Microsystem and Information Technology, Chinese Academy of Sciences, Shanghai 200050, China; guanghui_hu@mail.sim.ac.cn (G.H.); xxli@mail.sim.ac.cn (X.L.); 2 School of Microelectronics, University of Chinese Academy of Sciences, Beijing 100049, China;; 3 Vtran Tech (Chang Zhou) CO., Ltd., Shanghai 200135, China

**Keywords:** indoor navigation, pedestrian dead-reckoning, magnetic perturbation, decision tree

## Abstract

In pedestrian inertial navigation, multi-sensor fusion is often used to obtain accurate heading estimates. As a widely distributed signal source, the geomagnetic field is convenient to provide sufficiently accurate heading angles. Unfortunately, there is a broad presence of artificial magnetic perturbations in indoor environments, leading to difficulties in geomagnetic correction. In this paper, by analyzing the spatial distribution model of the magnetic interference field on the geomagnetic field, two quantitative features have been found to be crucial in distinguishing normal magnetic data from anomalies. By leveraging these two features and the classification and regression tree (CART) algorithm, we trained a decision tree that is capable of extracting magnetic data from distorted measurements. Furthermore, this well-trained decision tree can be used as a reject gate in a Kalman filter. By combining the decision tree and Kalman filter, a high-precision indoor pedestrian navigation system based on a magnetically assisted inertial system is proposed. This system is then validated in a real indoor environment, and the results show that our system delivers state-of-the-art positioning performance. Compared to other baseline algorithms, an improvement of over 70% in the positioning accuracy is achieved.

## 1. Introduction

Indoor navigation refers to the technology that provides positioning and position tracking services in large buildings or other places with certain closed structures. Indoor navigation may be applied to various scenes, including urban fire rescue [1], elderly care in nursing homes [2], tracing a parked car in a parking lot [3], and personnel tracking in underground mines [4]. Due to the loss of satellite signals indoors, the popular Global Navigation Satellite System (GNSS) is ineffective for indoor positioning. Therefore, a new high-precision indoor positioning technique is urgently needed. At present, passive wireless signal positioning technology and active inertial navigation technology are the two mainstream solutions [5,6].

A wireless signal-based positioning system relies on specific signal-emitting base stations. As a result, the accuracy and range of such positioning systems are heavily reliant on the numbers of base stations, which means that it may be financially costly to achieve a large-scale or high-precision implementation. Moreover, there are many challenges in obtaining accurate positioning through wireless signals due to the multipath effects and decay in obstacles [7,8,9]. It is also worth noting that if personal mobile phones are employed, users will have to face privacy problems involving personal information security [10].

In contrast to wireless positioning systems, pedestrian dead-reckoning (PDR) systems make use of 9 degrees of freedom (DOF) inertial measurement units (IMUs), which are composed of a magnetometer, an accelerometer, and a gyroscope, and are attached to the human body to estimate a person’s relative location. Therefore, PDR systems require little or no infrastructure to be pre-installed in buildings [11]. PDR employs inertial sensors to obtain the step lengths and directions of walking pedestrians. PDR is also autonomous and insusceptible to external jamming. Even when using a mobile phone as the terminal, there are no extra security concerns. With the maturity of MEMS technology, IMUs can be mass-produced; they are becoming progressively smaller and of a lower cost. Currently, IMUs are widely deployed in smartphones and wearable devices and headset [12]. There are a growing number of researchers engaged in related research topics [13,14,15].

Pedestrian Navigation Systems based on IMUs can be achieved through obtaining the relative attitude angles, absolute heading angles, and motion accelerations of the carriers by gyroscopes, magnetometers, and accelerometers, respectively. IMU sensors suffer from various noise, such as gyro offset instability, angular velocity, and random walking. Fortunately, the heading angle can be calculated based on sensor data fusion by utilizing the advantages of different sensors. During the navigation process, ferromagnetic structures inside indoor environments cause interference in geomagnetic fields, thereby corrupting magnetic data quality. Several researchers have made various attempts to address the problems mentioned above. An effective technique called Zero Velocity Update (ZUPT) was used in [16]. This algorithm increases the accuracy of heading angles by correcting the offset of the gyroscope and accelerometer through the time interval of foot landing. Another paper [17] verified that placing IMUs on the waist is the most beneficial to reduce the deviation between gyroscope measurements and the true value of the heading angle. These efforts are limited in reducing the measurement errors of sensors and do not consider the heading angle errors caused by the magnetic anomalies.

Indoor magnetic fields may be severely distorted due to the existence of magnetic perturbations in buildings. Thus, the heading angles cannot be correctly calculated by magnetic data directly. Therefore, researchers have proposed different approaches to optimize the magnetic data process and obtain true geomagnetic data. In [18], the authors placed multiple magnetic sensors in a circle to capture the magnetic fields in all directions, and perturbation was treated as a dipole magnetic field; using a complicated model, the perturbation was then subtracted from the uniform geomagnetic field. However, this method is complex and its precision is limited by the numbers of sensors. The study in [19] proposed that installing IMUs on the upper body can avoid interference from cables laid underground and other surface magnetic interference sources. However, magnetic anomalies originating from sources like steel structures cannot be properly handled by this method. Researchers adopted the Quasi Static Field (QSF) method to obtain magnetic data without interference by using a Generalized Likelihood Ratio Test (GLRT) in [20,21,22]. The false alarm probability can be high when using GLRT, which is based on a single threshold.

In recent years, machine learning has become a very important technology with broad application prospects [23,24]. One of the most commonly used data classification methods is a decision tree algorithm. Compared with other machine learning methods like a Deep Convolutional Network (DCN), decision tree is intuitive and fast and is computationally efficient and suitable to be integrated into a real-time pedestrian inertial navigation system. Its structure is similar to a tree prediction model, in which internal nodes represent tests of attributes and the end nodes represent the final classification results. This splits the complicated decision process into a series of simple choices and more accurately represents the process of human decision making. During the learning phase, the decision tree model is established by using training data to pursue the minimization of a given loss function. In the predicting phase, new data are classified by the trained decision tree model. These decision trees mainly use three methods: the ID3 algorithm proposed by Quinlan in 1986, the C4.5 algorithm proposed in 1993, and the CART algorithm proposed by Breiman et al. in 1984 [25]. As decision tree algorithms are able to provide satisfying results for large data sets over a short training time at the cost of relatively low computational complexity, they can be used in the navigation and positioning process, which inherently requires fast computation [26,27].

In this paper, we proposed a method to classify the magnetic field conditions in indoor navigation using a decision tree. By applying a rotation invariance of the geomagnetic field modulus, we analyzed the measurement data to obtain the characteristics of the magnetic field’s interference and discovered two features that can be utilized in the decision tree for the classification process. After being trained with experimental data, the decision tree can be used to find the true geomagnetic fields. Then, the Kalman Filter is used to calculate the heading by fusing the gyroscope and processed magnetic data.

The rest of this paper is organized as follows: Section 2 studies the Earth’s magnetic fields and compares indoor magnetic data with outdoor data to analyze the characteristics of the disturbed magnetic field. We then train a decision tree using the indoor data and extract the undisturbed magnetic data by the classification results. In Section 3, by combining the decision tree with a Kalman filter, a new data fusion method is proposed to estimate the heading angle. Section 4 focuses on experimental analyses. Finally, the conclusions are presented in Section 5.

## 2. Classification of Magnetic Data with a Decision Tree

### 2.1. Geomagnetic Information Analysis

**Figure 1:**
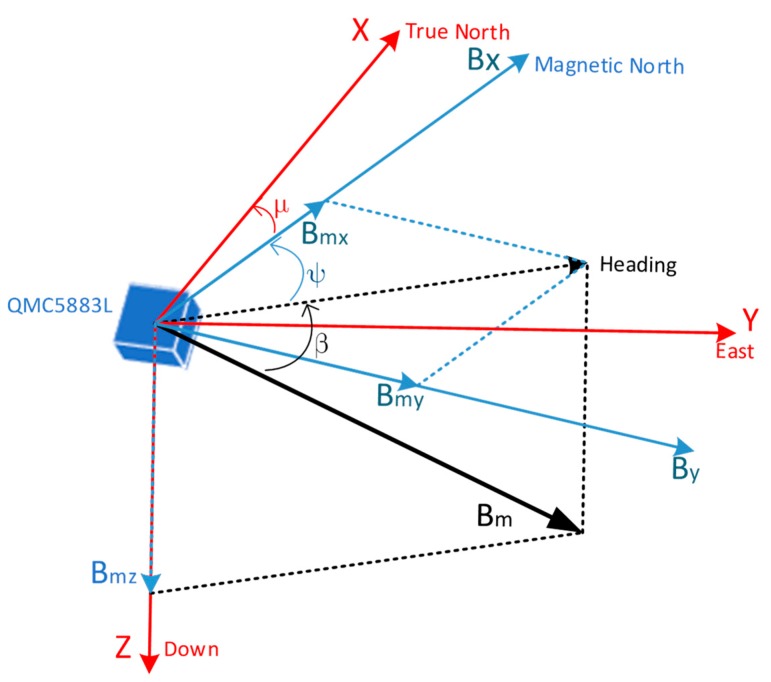
Schematic diagram of the geomagnetic signal model analysis.

The Earth acts like a giant magnet, with the line from the South Pole to the North Pole forming a magnetic meridian. In most places, the heading can be provided by an electronic compass, as long as we know the declination μ between the local longitude line and magnetic meridian as well as the inclination β, which is between the vector direction of geomagnetic field $B_{m}$ and the horizontal plane. The distribution of the geomagnetic field varies extremely slowly over time and space. When the carrier moves in a small space, $B_{m}$, β, and μ are considered constants that can be found in the geomagnetic field model WMM2020 [28] according to local latitude and longitude. As shown in Figure 1, the three-axis HMC5883L magnetic sensor, whose sampling frequency is set to 66.7 Hz, is used to measure the three-axis geomagnetic field information $B_{mx}$, $B_{my}$, and $B_{mz}$ and to calculate the magnetic heading angle ψ. Finally, the geographical heading angle of the carrier is obtained by a compensation calculation.

**Figure 2:**
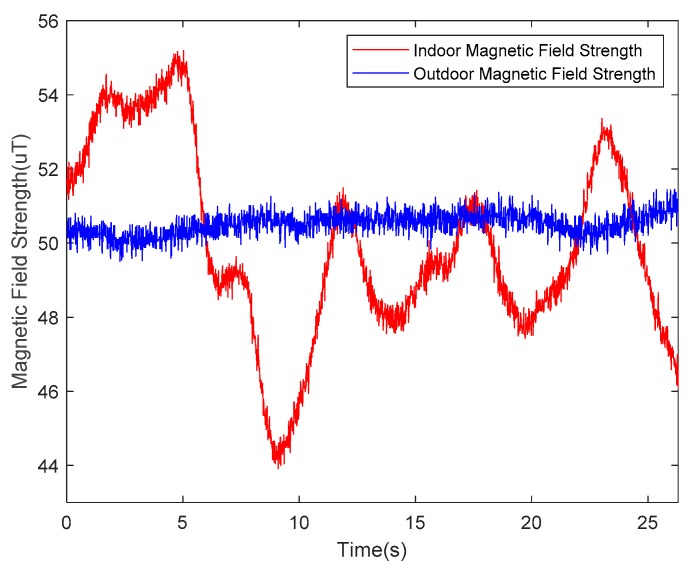
The measurements of the geomagnetic field. The red line is obtained indoors, and the blue line is obtained outdoors.

The undisturbed geomagnetic field signal can provide a precise heading angle for a carrier platform. The magnetic fields in some indoor environments, however, are seriously disturbed by various ferro material structures or power lines. Examples of disturbance sources are steel structures in the building, buried wires in the walls and floors, iron doors, windows, and other ferromagnetic materials or electric devices, such as elevators. Among these numerous magnetic interference sources, some interference sources are fixed (e.g., doors and windows), while others are varied, such as elevators and the current in the power lines. These factors make magnetic fields even more complicated. According to the WMM2020 model, the magnetic field strength can be considered as a constant in a small area of the Earth’s surface. As indoor navigation is usually applied in a building complex, the approximation is satisfied. The selected nearby outdoor space can be considered as a clean environment compared to the indoor space, as shown in Figure 2, where the blue line represents the outdoor magnetic field strength, and the red line represents its indoor counterpart. Clearly, extra steps are needed for indoor magnetic fields to extract useful information from these distractions.

### 2.2. Decision Tree for Extracting geomagnetic Field Data

A decision tree is a data processing method used in classification tasks. This tree represents the process of classifying instances based on features, which can be considered an if–then set and a conditional probability distribution based on features and classes. The advantages of this method mainly include the intuitiveness of the model, its fast classification speed, and its small consumption of computing resources, all of which benefit pedestrian inertial navigation systems.

In order to train a decision tree that can identify magnetic anomalies accurately, we need to analyze the magnetic field measurements and choose reliable features to build up. The vector form of the geomagnetic field can be obtained by a three-axis magnetic sensor, and the modulus of the vector can be regarded as constant in the indoor navigation area without measurement errors. This phenomenon is known as rotation invariance [29], in which the modulus of the geomagnetic field data on the three-axis magnetic sensor does not change with the attitude angles. However, there are calibration errors, systematic errors, and measurement errors in magnetic sensor measurements. The systematic errors of magnetic sensors can be mitigated by magnetic sensor calibration methods [30,31,32]. However, the data after calibration still contain noise, comprising intrinsic sensor noise and geomagnetic noise. Assuming that these errors obey a Gaussian distribution, the modulus measured by the magnetic sensor is shown in Equation (1). B represents the true value of the geomagnetic field strength, and $B_{0}$ represents the observed value of the geomagnetic field strength. ε defined in the braces represents the random error of measurement, which is Gaussian white noise according to the discussion above. it obeys distribution probability function P and its power spectral density is noted as $R_{\varepsilon }$. $N_{0}$ is a positive real constant which is used to describe the spectrum energy of the White Noise. Thus, at a certain time point $t_{i}$, the value of $\varepsilon (t_{i})$ is calculated according to Gaussian distribution, whose given probability of $\varepsilon (t_{i}=x)$ is P:

(1) $$B_{0}(t_{i})=B(t_{i})+\varepsilon (t_{i}),\{\begin{array}{c} P(\varepsilon (t_{i})=x)=\frac{1}{\sqrt{2\pi }\sigma }\exp(-\frac{x^{2}}{2\sigma^{2}})\\ R_{\varepsilon }(\tau =t_{i}-t_{j})=\frac{N_{0}}{2}\delta (\tau ) \end{array}$$

The magnetic data collected by the magnetic sensors in an outdoor interference-free environment satisfy the Gaussian distribution model. However, the indoor magnetic disturbances are extremely unevenly distributed and randomly scattered in every axial direction. As a result, the geomagnetic field vector is disturbed, and the calculated heading angle is biased. The observed value $B_{1}$ thus shows strong random fluctuations rather than maintaining rotation invariance.

(2) $$B_{1}(t_{i})=B(t_{i})+\eta (t_{i})+\varepsilon (t_{i}),\{\begin{array}{c} P(\varepsilon (t_{i})=x)=\frac{1}{\sqrt{2\pi }\sigma }\exp(-\frac{x^{2}}{2\sigma^{2}})\\ R_{\varepsilon }(\tau =t_{i}-t_{j})=\frac{N_{0}}{2}\delta (\tau ) \end{array}$$

where η represents the stochastic magnetic disturbances, which are the sum of the various magnetic disturbances. As mentioned before, the geomagnetic field data obeys a Gaussian distribution without external interference, while the magnetic data disturbances are most likely no longer Gaussian. Therefore, we can convert the detection of magnetic anomalies into the detection of non-Gaussian distributed magnetic data. We will explain how to employ the decision tree algorithm to address this problem in the following.

First, it is important to clarify that the classification object is a sequence of data like $ℬ=\left\{B_{1},B_{2},B_{3},\cdots ,B_{n}\right\}$, where n represents the number of data points, and $B_{i}\left(i=1,2,3,\cdots ,n\right)$ is the magnetic vector modulus measured by the magnetometer at each data point. If the measurement of the magnetic field is not disturbed, the deviation between the data and the true value ℬ is caused by the Gaussian error model of the measurement. As a result, the deviation will fluctuate within a certain range, as well as the measurement.

However, if the magnetic data are disturbed by local interference, the magnetic field data will no longer belong to a Gaussian distribution. The interference will be strengthened when approaching the source of the interference, and it will be weakened when away from the interference source. The mean value will present a notable deviation with respect to the original. Therefore, a method is proposed to evaluate the change of the mean in a set of measurement data by consistency. The consistency C of the data represents the notion of data consistency, which is obtained by Equation (3):

(3) $$C=\frac{\mathrm{mean}(ℬ1)-\mathrm{mean}(ℬ2)}{\sigma }$$

where ℬ is a set of magnetic field data, ℬ1 is a subset of ℬ, which consists of the first half elements of ℬ, and ℬ2 is a complement of ℬ1 in ℬ. The mean of ℬ1 and ℬ2 can be considered as two numbers in the mean of the measurement value, and C will obey the distribution of N(0,2). The probability density function of C is given as:

(4) $$P(\left|C\right|<X)=\int_{-X<x<X}\frac{1}{2\sqrt{\pi }}\exp(-\frac{x^{2}}{4})dx$$

Therefore, if the data distribution of ℬ obeys a Gaussian distribution, then there is a 95.4% probability that C falls into the interval $\left(-2\sqrt{2},2\sqrt{2}\right)$. According to the Bayesian estimation principle, we take the value of C as the observation basis. If the value of C falls into the interval $\left(-2\sqrt{2},2\sqrt{2}\right)$, it can be determined that ℬ obeys a Gaussian distribution, and the confidence will be (1–4.6%). Therefore, we chose C as the first feature of the decision tree criterion, as C can be used to determine the possibility that ℬ obeys a Gaussian distribution.

Interference η also introduces fluctuations, thereby enlarging the dynamic range of the data. The mean of the measurement data may not change significantly; therefore, the distribution of the measurements will no longer maintain a Gaussian distribution. Based on this, we introduce the concept of fluctuation. We define the fluctuation F of the data as the difference between the maximum and minimum value divided by σ; F reflects the fluctuation of the data series ℬ and is given by:

(5) $$F=\frac{\max(B)-\min(B)}{\sigma }$$

For a set of undisturbed magnetic field measurement data, ℬ should obey an n-dimensional joint Gaussian distribution. The distribution function of F can then be obtained by:

(6) $$\begin{array}{c} P(\left|F\right|<X)=\int_{\Omega }dP(B_{1},B_{2},\ldots B_{n})\\ \Omega :\{(B_{1},B_{2},\cdots ,\;B_{n})\;|\;\left|B_{i}-B_{j}\right|<X,i\neq j,i,j=0,1,2,\cdots ,n)\} \end{array}$$

Based on the calculation, F clearly obeys a Gaussian distribution, whose variance is n. If the data in ℬ meet the requirements of Gaussian distribution, there will be a 95.4% probability that F falls into the interval $\left(-2\sqrt{n},2\sqrt{n}\right)$. According to the Bayesian principle, if we take the value of F as the observation basis, when the value of F falls into the interval $\left(-2\sqrt{n},2\sqrt{n}\right)$, we can make an assumption that ℬ obeys Gaussian distribution, and the confidence is (1–4.6%). Therefore, we choose F as the second feature of the decision tree.

Based on aforementioned two indicators and a data sequence with a length of 15, a decision tree that identifies magnetic anomalies can be trained. In the learning process, the decision tree model is established by using the training data set and minimizing the loss function. During prediction, the new data are classified by the decision tree model. In this paper, we use a Classification and Regression Tree (CART) based on the Gini Index Minimization Criteria (GIMC) for data classification. Assuming that K is the number of total classes and the probability that class k is $p_{k}$, the expression of the Gini coefficient is:

(7) $$\mathrm{Gini}(p)=\sum_{k=1}^{K}p_{k}(1-p_{k})=1-\sum_{k=1}^{K}p_{k}^{2}$$

Magnetic anomaly identification is a two-class problem that deals with both Gaussian and non-Gaussian distributions. The Gini coefficient expression can be simplified as:

(8) Gini(p)=2p(1−p)

For a given set of magnetic data samples D, the samples can be classified into two classes, $D_{1}$ and $D_{2}$, according to a certain standard of the eigenvalue A:

(9) $$D_{1}=\{(x,y)\in D|A(x)=a\},\;D_{2}=D-D_{1}$$

The expression of D’s Gini coefficient is:

(10) $$\mathrm{Gini}(D,A)=\frac{\left|D_{1}\right|}{\left|D\right|}\mathrm{Gini}(D_{1})+\frac{\left|D_{2}\right|}{\left|D\right|}\mathrm{Gini}(D_{2})$$

**Figure 3:**
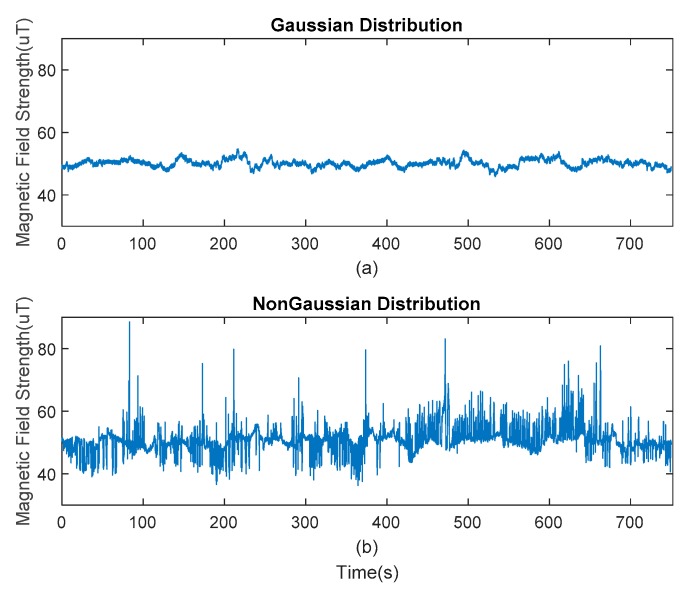
Magnetic field observations under different conditions: (**a**) an interference-free condition and (**b**) an interference condition.

In order to verify the proposed decision tree algorithm, first, the data set must be collected. The testers wearing the IMUs on their waists collected magnetic data in a magnetic-clean open space that can be treated as a baseline magnetic environment. As expected, the collected data follow a Gaussian distribution due to rotational invariance. In the same environment, the disturbed data set is collected by introducing artificial magnetic sources to simulate the magnetic disturbances in the room. Figure 3a shows the undisturbed magnetic data, and Figure 3b shows the magnetic data under the condition of simulated indoor irregular magnetic interference. These data are used to train the decision tree and generate the model parameters.

**Figure 4:**
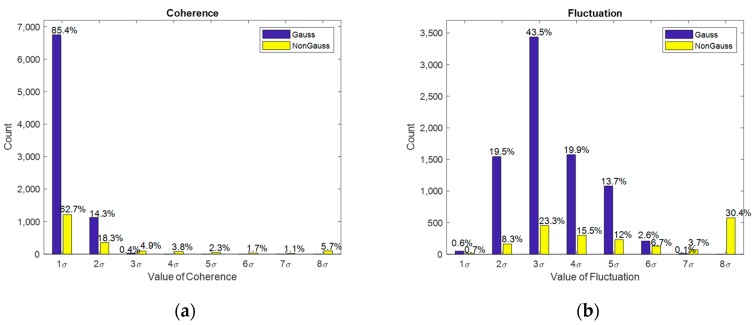
Statistical distribution of the two features. (**a**) Probability distribution of consistency. Blue bars indicate the values for Gaussian distribution, and the yellow bars indicate the value for non-Gaussian distribution. (**b**) Probability distribution of fluctuation. Blue bars indicate the values for Gaussian distribution, and yellow bars indicate the values for non-Gaussian distribution.

In Figure 4a, the blue bars represent the consistency distribution of the Gaussian data sets and 99.7% of the data distributed within 2σ. On the other hand, the yellow bars represent the consistency distribution of the non-Gaussian data-sets under the influence of abnormal magnetic field interference sources; here, the consistency distribution is clearly more scattered than the magnetic data with no interference. In Figure 4b, the blue bars show the distribution of the Gaussian data set, and 99.9% of the data are distributed within 6σ. In contrast, the yellow bars show the volatility distribution of the non-Gaussian data set, and the scattered data distribution is influenced by external magnetic interference sources.

The collected data are classified based on interference and non-interference. As shown in Table 1, the fluctuation value, the consistency value, and the Gaussian and non-Gaussian categories are listed relative to the numbering of the data group and are used for decision tree training. The data group size was varied and optimized for modeling accuracy.

**Figure 5:**
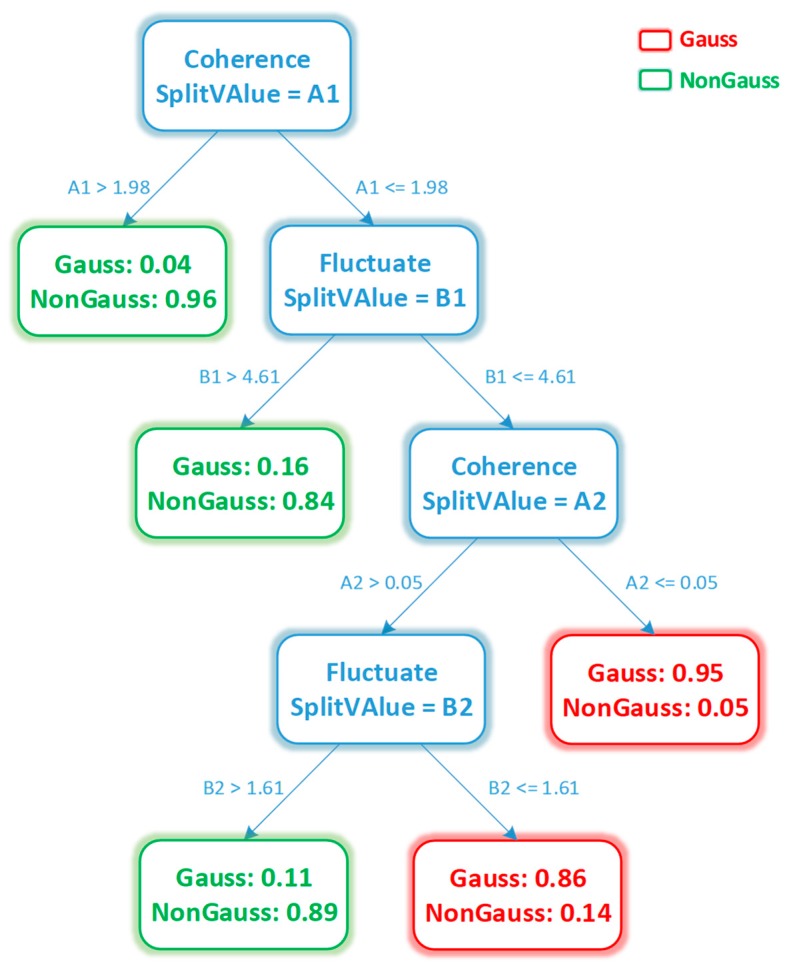
Tree diagram for the decision tree. A stands for the consistency feature, and B stands for the fluctuation feature. The red end nodes indicate that the input data are Gaussian, while the green end nodes mean that the input data are non-Gaussian.

In order to avoid over-fitting by excessive layers of the decision tree, the maximum split layer number is set to 4. The model is also cross-validated by 10-fold cross-validation. The decision tree generation graph is shown in Figure 5. The red cotyledons indicate the Gaussian class, and the green cotyledons indicate the non-Gaussian class. The fluctuation features eventually adopt the eigenvalues B1 and B2, and the consistency features adopt eigenvalues A1 and A2.

**Figure 6:**
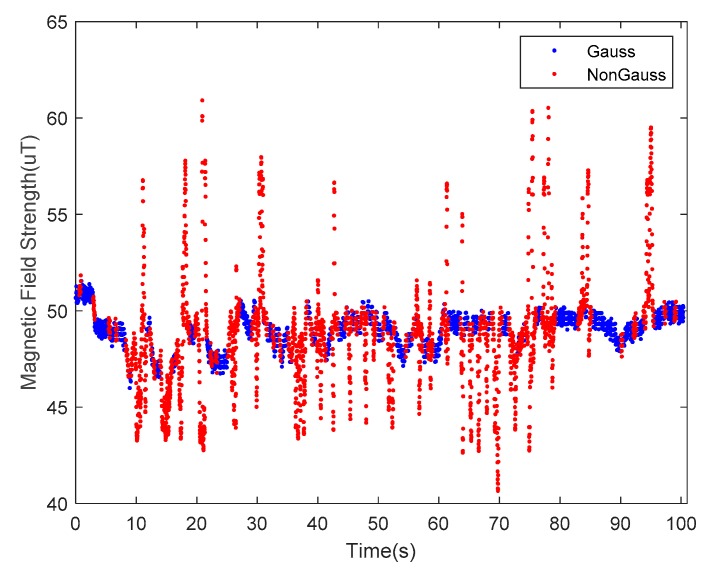
A decision tree extracts interference-free magnetic data from the measurement data of the magnetometer.

Using the generated decision tree to predict the test data set can verify the precision of the predictive classification. We extracted a data-set containing disturbed magnetic data and verified the classification results by using a decision tree. We obtained this decision tree with the collected interference data and non-interference data. The first node shows that if the value of coherence is large enough, it can be used to screen non-Gaussian data with high confidence. When the value of coherence is not enough to screen out the Gaussian data, we can filter the Gaussian data according to the fluctuation value at different value levels of coherence. In this way, we can generate other nodes of the decision tree. Figure 6 shows the results of decision tree classification in magnetic data sets with interference. The blue data points in the figure represent the magnetic data with Gaussian distribution during data acquisition, and the red data points represent the disturbed magnetic data with non-Gaussian distribution. We found that the accuracy of the decision tree classification was as high as 92% for the sample classification. 

The generated decision tree can detect whether a data sequence obeys Gaussian distribution, which does not depend on the position where the data is obtained and only relies on the sequential relationships between data points. Considering the environment-independent nature of the data’s statistical features, a decision tree can be used to work well in other environments. Geomagnetic field data can be obtained through filtering and can be fused into the Kalman filter to calculate the heading angle.

## 3. Heading Algorithm Based on a Decision Tree and the Kalman Filter

### 3.1. Attitude Angle Error Equation

A Kalman filter based on multi-sensor fusion is one of the most effective methods to calculate attitude angles. The Kalman algorithm is an optimal linear filtering method based on the minimum variance principle. It estimates the attitude angles through state equations and uses observation equations to correct those estimates. Since the Kalman filter is a filter method in the time domain, this calculation is a continuous loop of prediction and correction. It does not need to store a large amount of data to solve equations, and the calculation is relatively simple, which satisfies the requirements of real-time navigation. The direct and indirect estimation methods can be used for the attitude angle estimation. The “direct method” means that the estimated object is the attitude angle of the system (i.e., the heading angle, pitch angle, and roll angle). The “indirect method” refers to estimating the errors of the attitude angles first and then using the errors to correct the angle estimates. The latter is used in this paper. After first-order approximation, the equations are linear in the indirect method, and a linear Kalman filter estimation can be directly employed to reduce computational complexity.

In the Kalman filter equations, attitude angle errors are set to be the state vector. It is assumed that the true attitude angle is composed of the angle estimation and the error of estimation during pedestrian walking. The real-time attitude also needs to be converted from the carrier’s coordinate system to a navigation coordinate system. Using the quaternion theory, the rotation process can be expressed in the following form:

(11) $$q_{b}^{n}(t)={\hat{q}}_{b}^{n}(t)⊗\delta q_{b}^{n}(t)$$

In Equation (11), the $q_{b}^{n}(t)$ represents the true value of the current attitude quaternion, ${\hat{q}}_{b}^{n}(t)$ represents the estimation, and $\delta q_{b}^{n}(t)$ represents the error of estimation. ⊗ is the quaternion multiplication symbol. The following equation can be obtained by the simultaneous differential on both sides of Equation (11):

(12) $${\dot{q}}_{b}^{n}(t)={\dot{\hat{q}}}_{b}^{n}(t)⊗\delta q_{b}^{n}(t)+{\hat{q}}_{b}^{n}(t)⊗\delta {\dot{q}}_{b}^{n}(t)$$

which is determined by the nature of the quaternion:

(13) $${\dot{q}}_{b}^{n}(t)=\frac{1}{2}q_{b}^{n}(t)⊗\omega_{nb}^{b}(t)$$

where $\omega_{nb}^{b}(t)=\omega_{ib}^{b}(t)-(\omega_{ie}^{b}(t)+\omega_{en}^{b}(t))$. $\omega_{ib}^{b}(t)$ is the carrier angular rate measurement, $\omega_{ie}^{b}(t)$ is the Earth rate measurement in the inertial frame, $\omega_{en}^{b}(t)$ is the navigation frame rate measurement in the Earth frame, and $\omega_{nb}^{b}(t)$ is the rotation angular rate measurement in the n-frame. In pedestrian inertial navigation, $\omega_{en}^{b}(t)$ and $\omega_{ie}^{b}(t)$ can be ignored since a consumer-level MEMS gyroscope is not sensitive to them. Therefore, the previous equation can be simplified to $\omega_{nb}^{b}(t)\approx \omega_{ib}^{b}(t)$, in which ${\hat{\omega }}_{nb}^{b}(t)$ represents the estimated value of $\omega_{nb}^{b}(t)$. At the same time, Equation (13) is brought into Equation (12), yielding

(14) $$2\delta {\dot{q}}_{b}^{n}(t)=\delta q_{b}^{n}(t)⊗\omega_{nb}^{b}(t)-{\hat{\omega }}_{nb}^{b}(t)⊗\delta q_{b}^{n}(t)$$

Since the error of $\delta q_{b}^{n}$ is small, it can be rewritten as $\delta q_{b}^{n}={\left[\begin{array}{cc} 1 & \delta q_{b}^{n} \end{array}\right]}^{T}$ according to the quaternion property. At the same time, the angular velocity can be rewritten into a quaternion vector form, $\omega_{nb}^{b}={\left[\begin{array}{cc} 0 & \omega_{nb}^{b} \end{array}\right]}^{T}$. Furthermore, Equation (14) can be expanded into a vector form:

(15) $$2\left[\begin{array}{c} \dot{1}\\ \delta {\dot{q}}_{b}^{n}(t) \end{array}\right]=\left[\begin{array}{c} 1\\ \delta q_{b}^{n}(t) \end{array}\right]⊗\left[\begin{array}{c} 0\\ \omega_{nb}^{b}(t) \end{array}\right]-\left[\begin{array}{c} 0\\ {\hat{\omega }}_{nb}^{b}(t) \end{array}\right]⊗\left[\begin{array}{c} 1\\ \delta q_{b}^{n}(t) \end{array}\right]$$

The quaternion operation rule gives:

(16) $$2\left[\begin{array}{c} \dot{1}\\ \delta {\dot{q}}_{b}^{n}(t) \end{array}\right]=\left[\begin{array}{c} \delta q_{b}^{n}(t)\cdot \omega_{nb}^{b}(t)-{\hat{\omega }}_{nb}^{b}(t)\cdot \delta q_{b}^{n}(t)\\ \delta q_{b}^{n}(t)\times \omega_{nb}^{b}(t)-{\hat{\omega }}_{nb}^{b}(t)\times \delta q_{b}^{n}(t)+\omega_{nb}^{b}(t)-{\hat{\omega }}_{nb}^{b}(t) \end{array}\right]$$

Omitting high-order small items, we obtain

(17) $$\delta {\dot{q}}_{b}^{n}(t)=-\left[{\hat{\omega }}_{nb}^{b}(t)\times \right]\delta q_{b}^{n}(t)+\frac{1}{2}b_{\omega }(t)$$

where $\left[{\hat{\omega }}_{nb}^{b}(t)\times \right]$ is the antisymmetric matrix, and $b_{\omega }(t)=\omega_{nb}^{b}(t)-{\hat{\omega }}_{nb}^{b}(t)$, indicating the estimated value of the gyro’s zero deviation at time t. Then, we construct the state vector, as follows:

(18) $$X=\left[\begin{array}{c} \delta q\\ b_{\omega } \end{array}\right]$$

Then, the state equation of the Kalman filter can be obtained by differentiating the state’s quantity:

(19) $$\dot{X}(t)=\left[\begin{array}{cc} -\left[{\hat{\omega }}_{nb}^{b}(t)\times \right] & \frac{1}{2}I_{3\times 3}\\ 0_{3\times 3} & 0_{3\times 3} \end{array}\right]X(t)+\left[\begin{array}{c} \frac{1}{2}W_{1}\\ W_{2} \end{array}\right]$$

where $b_{\omega }$ represents the gyro bias, which includes random noise, $W_{1}$, and $b_{\omega }$ is the first-order autoregressive model. The system sampling frequency is much higher than the change rate of the gyro zero drift. The random walk model can be used instead to meet the accuracy requirements. Suppose ${\dot{b}}_{\omega }=W_{2}$, and $W_{2}$ is a zero-mean noise with low intensity. In this way, the equation for the state, according to the relationship between state quantity and noise, is attained.

### 3.2. Observation Equation Using Magnetic Data from Decision Tree

The angular velocity of the gyroscope is used as a parameter to update the quaternion state in real time. Gyroscope data contain time-varying errors such as zero-bias instability, which causes the measurement accuracy to decrease with time. It is necessary to correct this deviation by fusing the geomagnetic field data. A decision tree can be used to screen the magnetometer data, and classified interference-free magnetic data are used as the observation values for heading angle correction to effectively improve the accuracy of the attitude angles.

Assume that $B_{DT}^{n}$ is the magnetic data with no interference, whose value can be converted by the carrier observation data as follows:

(20) $$B_{DT}^{n}(t)=q_{b}^{n}⊗B_{DT}^{b}(t)⊗q_{b}^{n}{}^{*}$$

There are observation errors in the magnetic data and, consequently, errors in the quaternion estimations. The error model of the observation equation is given as follows:

(21) $$\delta B_{DT}^{n}(t+1)=B_{DT}^{n}(t)-(\delta q_{b}^{n}⊗{\hat{q}}_{b}^{n})⊗({\hat{B}}_{DT}^{b}(t+1)+\delta B_{DT}^{b}(t+1))⊗{\left(\delta q_{b}^{n}⊗{\hat{q}}_{b}^{n}\right)}^{*}$$

Simplifying the error equation and omitting the high-order infinitesimal yields:

(22) $$\delta B_{DT}^{n}(t+1)=B_{DT}^{n}(t)-\delta q_{b}^{n}⊗{\hat{B}}_{DT}^{n}(t+1)⊗\delta q_{b}^{n}{}^{*}$$

Expanding Equation (22) according to the quaternion operational rule yields:

(23) $$\begin{array}{cc} \; & \left[\begin{array}{c} 0\\ \delta B_{DT}^{n}\left(t+1\right) \end{array}\right]=\left[\begin{array}{c} 0\\ B_{DT}^{n}\left(t\right) \end{array}\right]-\left[\begin{array}{c} 1\\ \delta q_{b}^{n} \end{array}\right]⊗\left[\begin{array}{c} 0\\ {\hat{B}}_{DT}^{n}\left(t+1\right) \end{array}\right]⊗\left[\begin{array}{c} 1\\ \delta q{{}_{b}^{n}}^{*} \end{array}\right]\\ \; & =\left[\begin{array}{c} 0\\ B_{DT}^{n}\left(t\right) \end{array}\right]-\left(\left[\begin{array}{c} 0\\ {\hat{B}}_{DT}^{n}\left(t+1\right) \end{array}\right]⊗\left[\begin{array}{c} 1\\ \delta q_{b}^{n} \end{array}\right]-\left[\begin{array}{c} 0\\ 2{\hat{B}}_{DT}^{n}\left(t+1\right)\times \delta q_{b}^{n} \end{array}\right]\right)⊗\left[\begin{array}{c} 1\\ \delta q{{}_{b}^{n}}^{*} \end{array}\right]\\ \; & =\left[\begin{array}{c} 0\\ B_{DT}^{n}\left(t\right) \end{array}\right]-\left[\begin{array}{c} 0\\ {\hat{B}}_{DT}^{n}\left(t+1\right) \end{array}\right]⊗\left[\begin{array}{c} 1\\ \delta q_{b}^{n} \end{array}\right]⊗\left[\begin{array}{c} 1\\ \delta q{{}_{b}^{n}}^{*} \end{array}\right]+\left[\begin{array}{c} 0\\ 2{\hat{B}}_{DT}^{n}\left(t+1\right)\times \delta q_{b}^{n} \end{array}\right]⊗\left[\begin{array}{c} 1\\ \delta q{{}_{b}^{n}}^{*} \end{array}\right]\\ \; & =\left[\begin{array}{c} 2{\hat{B}}_{DT}^{n}\left(t+1\right)\times \delta q_{b}^{n}\cdot \delta q{{}_{b}^{n}}^{*}\\ 2\left[{\hat{B}}_{DT}^{n}\left(t+1\right)\times \right]\delta q_{b}^{n}+2\left[{\hat{B}}_{DT}^{n}\left(t+1\right)\times \right]\left[\delta q_{b}^{n}\times \right]\delta q{{}_{b}^{n}}^{*} \end{array}\right] \end{array}$$

Omitting the high-order infinitesimal gives:

(24) $$\delta B_{DT}^{n}(t+1)=2\left[{\hat{B}}_{DT}^{n}(t+1)\times \right]\delta q_{b}^{n}$$

If we differentiate the equation, we obtain:

(25) $$\delta {\dot{B}}_{DT}^{n}(t+1)=2\left[{\dot{\hat{B}}}_{DT}^{n}(t+1)\times \right]\delta q_{b}^{n}+2\left[{\hat{B}}_{DT}^{n}(t+1)\times \right]\delta {\dot{q}}_{b}^{n}$$

where ${\hat{B}}_{DT}^{n}$ is the estimated value, which can be treated as a constant. Equation (26) can be obtained by substituting Equation (17) into Equation (25):

(26) $$\delta {\dot{B}}_{DT}^{n}(t+1)=-2\left[{\hat{B}}_{DT}^{n}(t+1)\times \right]\left[\omega_{nb}^{b}\times \right]\delta q_{b}^{n}+\left[{\hat{B}}_{DT}^{n}(t+1)\times \right]b_{\omega }$$

Reorganizing Equations (24) and (26) yields the observation equation:

(27) $$\left[\begin{array}{c} \delta B_{DT}^{n}(t+1)\\ \delta {\dot{B}}_{DT}^{n}(t+1) \end{array}\right]=\left[\begin{array}{cc} 2\left[{\hat{B}}_{DT}^{n}(t+1)\times \right] & 0_{3\times 3}\\ -2\left[{\hat{B}}_{DT}^{n}(t+1)\times \right]\left[\omega_{nb}^{b}\times \right] & \left[{\hat{B}}_{DT}^{n}(t+1)\times \right] \end{array}\right]X+\left[\begin{array}{c} V_{1}\\ V_{2} \end{array}\right]$$

where $V_{1}$ and $V_{2}$ are observation errors that satisfy the Gaussian distribution.

**Figure 7:**
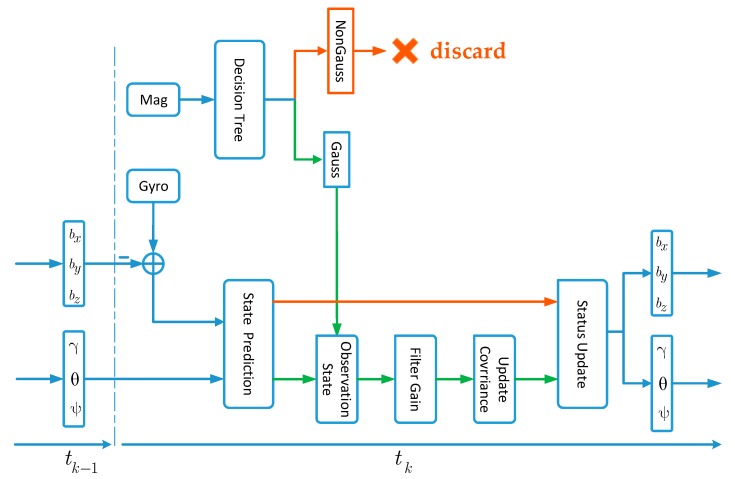
Flow chart of the proposed algorithm.

A flow chart for the attitude angle calculation is shown in Figure 7, among which γ represents the roll angle, θ represents the pitch angle, and ψ represents the heading angle. When the attitude angle is small, the error quaternion and error attitude angle can be transformed into each other. $b_{x}$, $b_{y}$, and $b_{z}$ are the zero offsets of the gyroscope’s three axes. Under the condition that the state vector of the previous time is known, the state vector of the current time can be predicted according to the state equation in combination with the observation of the gyroscope. In practical applications, the measured magnetic data are first handled by the decision tree, which can determine whether the magnetic dataset obeys a Gaussian distribution. When the decision considers the current magnetic dataset to be a Gaussian sequence, magnetic data can be used as the observation value to enter the Kalman filter to solve the attitude angle. When the decision tree classifies the magnetic dataset as a non-Gaussian sequence, the magnetic data are considered interference data and discarded, and the attitude angle is updated with calibrated gyroscope data. Since magnetic interference is local and gyro data has high accuracy in a short time, the proposed algorithm can utilize the gyro data to tolerate that the magnetic data is unavailable for a certain period in pedestrian inertial navigation.

## 4. Results and Discussion

### 4.1. Experimental Equipment and Test Environment

**Figure 8:**
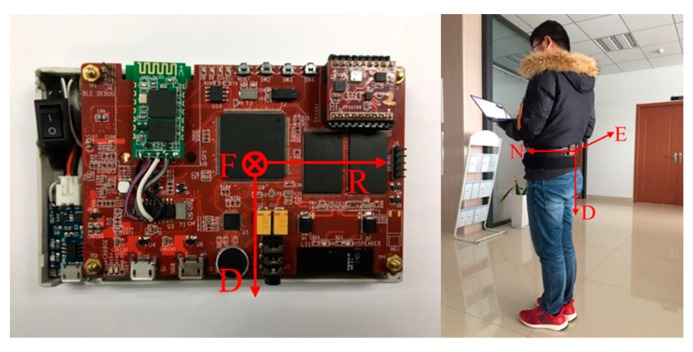
VT-IMUs wearable devices and installation.

A wearable IMUs device worn on the waist was used to test the navigation algorithm and verify the effectiveness of the magnetic data classification method. In Figure 8, the left picture shows the pedestrian inertial navigation system designed by our team. We call the system “VT-IMUs”. It is composed of consumer-level 9 DOF MEMS sensors, microprocessors, and other electronic components. The sampling frequency of the system is 66.7 Hz, and some parameters of the MEMS sensors are shown in Table 2. The sensor coordinate system is noted as *O*-*X*_s_*Y*_s_*Z*_s_, where the directions are set as “front–right–down”, and these three axes are coordinated with the axes of the gyroscope. When the VT-IMUs are worn on the pedestrian’s waist, coordinate systems of carriers and sensors are fixed. For convenience, it is assumed that the carrier coordinate system and sensor coordinate system are coincident. As shown in the right of Figure 8, the navigation coordinate system in this paper is set as the “North–East–Down” geographic coordinate system. 

**Figure 9:**
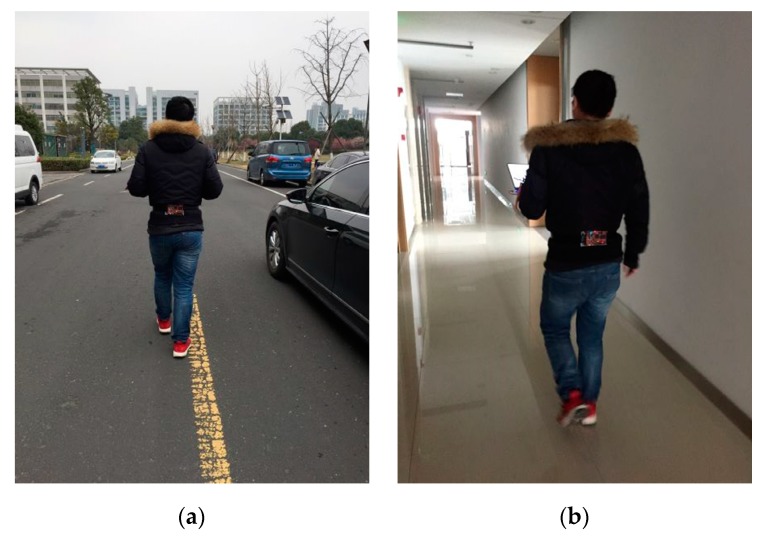
Test data collection in different scenes: (**a**) outdoor tests; (**b**) indoor tests.

In order to verify the performance of the decision tree, the urban road and interior of the office building were selected as the test environments. As shown in Figure 9, there are ferromagnetic materials on urban roads, such as fire hydrants, sewer covers, and vehicles, which will interfere with the measurement of the magnetometer. Compared with the outdoor magnetic environment, the indoor magnetic field distribution is more complicated since there are a large number of interference sources like steel structures, buried cables, doors and windows, etc.

### 4.2. Pedestrian Stride Estimate

In pedestrian inertial navigation, the pedestrian track is estimated by the heading angle and stride length. The estimation of a step’s stride mainly depends on the measurement data of the accelerometer. Many researchers have been working to improve the accuracy of step stride estimation. Methods for estimating step stride have thus been studied in the literature [33,34]. In this paper, the Weinberg method is used as per paper [35]. While walking, the knee is bent only when the foot is off the ground, and the leg can be treated as a lever of a fixed length while the foot is on the ground. Therefore, an empirical nonlinear model can be used to estimate the stride length:

(28) $$L=K\times \sqrt[{4}]{A_{\max}-A_{\min}}+b$$

where *L* is the stride length, and *A*_max_ (or *A*_min_) is the maximum (or minimum) modulus of acceleration in a step. *K* is a personalized parameter, and *b* is the correction factor used to reduce the estimate error.

Our pedestrian inertial navigation system is based on the Step-and-Heading method, which is recognized as a relative positioning algorithm. After initializing the position coordinates, the pedestrian’s position in real-time can be obtained through a location update. The navigation equation is as follows:

(29) $$\{\begin{array}{c} x_{k}=x_{0}+\sum_{i=1}^{k}L_{i}\cos\psi_{i}\\ y_{k}=y_{0}+\sum_{i=1}^{k}L_{i}\sin\psi_{i} \end{array}$$

where $\left(x_{k},y_{k}\right)$ represents the real-time location of the pedestrian, and $\left(x_{0},y_{0}\right)$ represents the starting position of the pedestrian, $\psi_{i}$ and $L_{i}(i=1,2,3,\cdots ,k)$ stands for the heading angle and stride length of step i, respectively. Thus, the pedestrian’s position can be calculated by (29) as soon as the parameters $\psi_{i}$ and $L_{i}$ are estimated.

### 4.3. Test and Discussion

As the heading angle and stride length are two key indicators, any error in them will affect the accuracy of navigation. This article only focuses on attitude estimation (the step length in Weinberg’s method (28)), which is necessary for de-correlating the stride error budget from the heading angle, thereby allowing the assessment of heading accuracies only.

**Figure 10:**
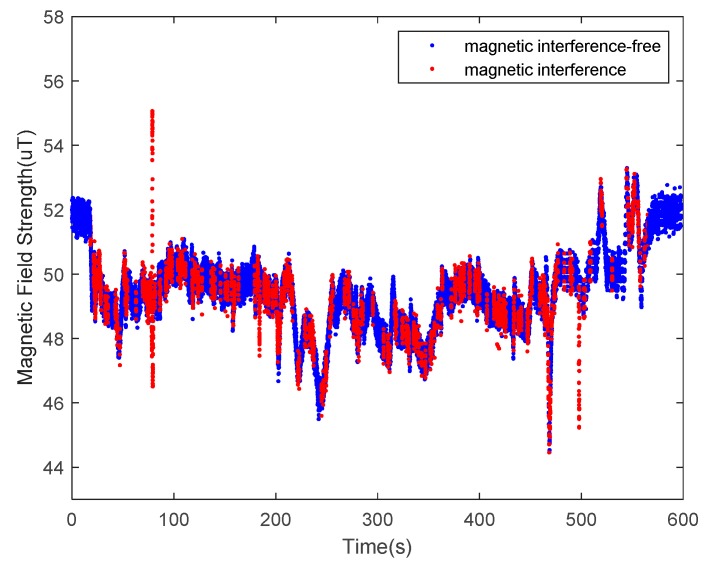
Magnetic data classification in outdoor pedestrian inertial navigation.

The performance of the decision tree-based magnetic classification algorithm was verified in an outdoor environment. The tester took a route on the test road and collected data for the VT-IMUs during the walking process. The magnetic data were classified by the trained decision tree, the result of which is shown in Figure 10. The blue dots represent the data selected by the decision tree that obey a Gaussian distribution, while the red dots represent the anomalies. The proportion of magnetic anomalies is 10.29%, indicating that there is little interference in the outdoor road environment.

**Figure 11:**
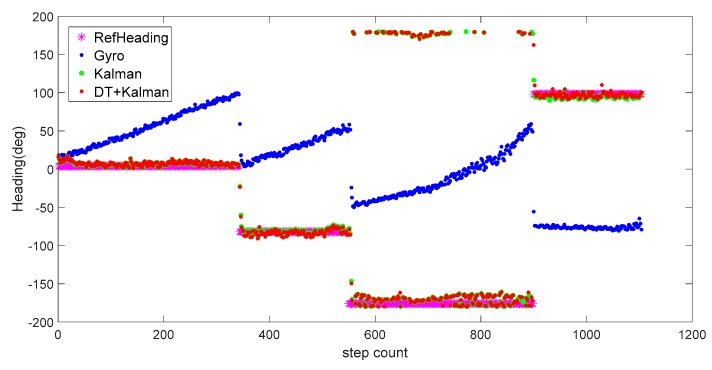
Heading angles for outdoor pedestrian inertial navigation.

To assess the effectiveness of the proposed algorithm in attitude angle calculation, we compared three calculation methods in the test. As shown in Figure 11, blue dots are the heading angles that were estimated by the gyro data directly, and the heading error accumulates gradually over time. Specifically, the horizontal axis represents the number of steps, and the vertical axis represents the heading angle corresponding to each step. Green dots are the heading angles calculated by the Kalman filter through a fusion of raw magnetic data and gyro data. It can be seen that the heading angle is significantly closer to the reference angle after fused with the outdoor magnetic data. Finally, the undisturbed geomagnetic data were screened from the original data by a decision tree and were combined with the Kalman filter to derive the heading angle. The red dots represent the result of the heading angle under the decision tree and Kalman (DT+Kalman) algorithm. The heading angle data obtained by the proposed method and the reference heading have a high degree of coincidence. Since there is less magnetic interference outdoors, the Kalman algorithm and proposed algorithm can achieve good heading angle estimations.

**Figure 12:**
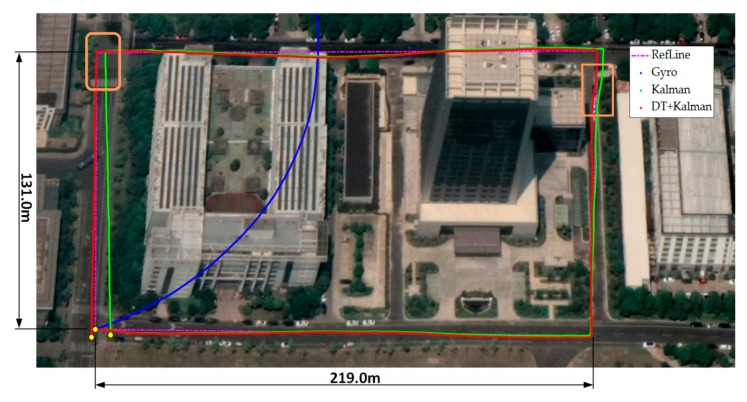
Trajectory test of the three different algorithms for outdoor pedestrian inertial navigation. The walking time is 598.6 s outdoors.

Figure 12 shows the tracking trajectory comparisons of one trace between the different heading estimate approaches. Clearly, the heading estimate error is the main localization error source for the pedestrian positioning trajectory. The error of the heading angle based on the gyro integral accumulates over time, and the test trajectory is far from the baseline trajectory. The proposed DT+Kalman method has the smallest heading estimation error; The method not only uses magnetic data to improve the accuracy of heading angle but also prevent the errors caused from abnormal magnetic anomalies. Thus, it obtains the best tracking performance. The orange box in upper right corner shows that the proposed algorithm can effectively mitigate the influence of magnetic interference in the heading angle, and the orange box in the upper left corner show that, without the correction from the magnetic data screened by our algorithm, the trajectory based on Kalman method will have an error in the horizontal coordinate due to the accumulation of inertial navigation errors.

Comparisons of the three different sets of trajectories are shown in Table 3. These trajectories are obtained by initializing the starting position and orientation for each loop, and the end location errors are caused only by the methods. The start and end positions are marked in yellow in Figure 12. By analyzing the Kalman method and the Gyro method, geomagnetic filed data play an important role in the heading angle deviation. Using the decision tree to eliminate magnetic data anomaly interference, the proposed DT+Kalman method further reduces the positioning error by 52.6%.

**Figure 13:**
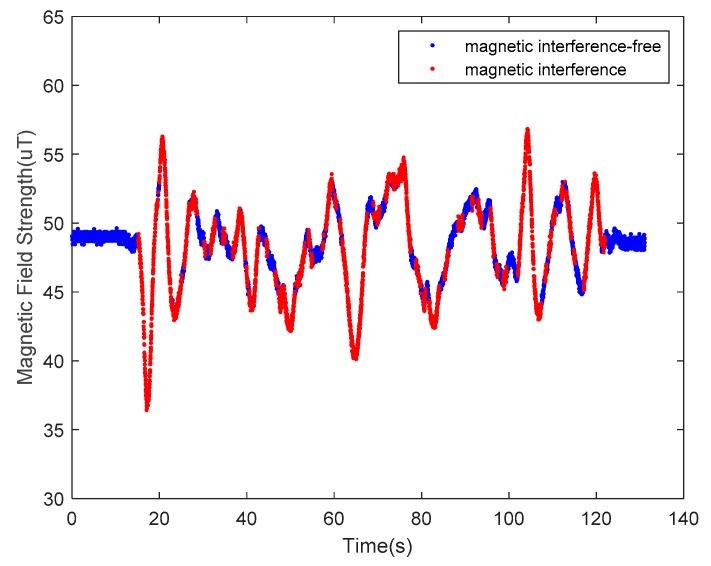
Magnetic data classification results generated by the decisions for indoor pedestrian inertial navigation. The red points are used to mark the interference measurements.

The indoor magnetic environments are much more complicated than the outdoor environments. As shown in Figure 9b, a typical office building was selected as our experimental site, including the office areas and floor aisles. The tester wearing the VT-IMUs collected data while walking. The distribution of magnetic data during this pedestrian walking cycle was collected, and the results are shown in Figure 13. The blue dots indicate the undisturbed measurements, and the red dots represent the disturbed data. The results show that the proportion of magnetic anomalies ranged up to 45.99%, indicating that the indoor interference was serious.

**Figure 14:**
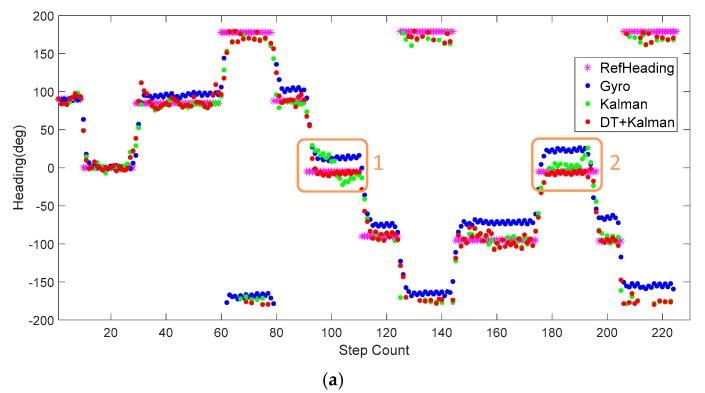

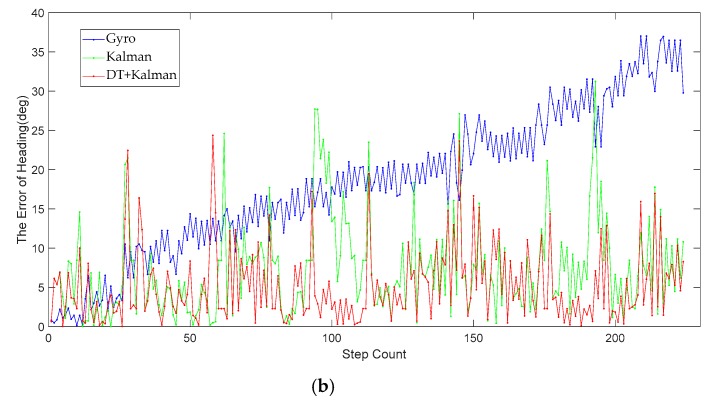
(**a**) The heading calculations of the three different algorithms for indoor pedestrian inertial navigation. (**b**) The error of the heading angle is based on the three algorithms.

**Figure 15:**
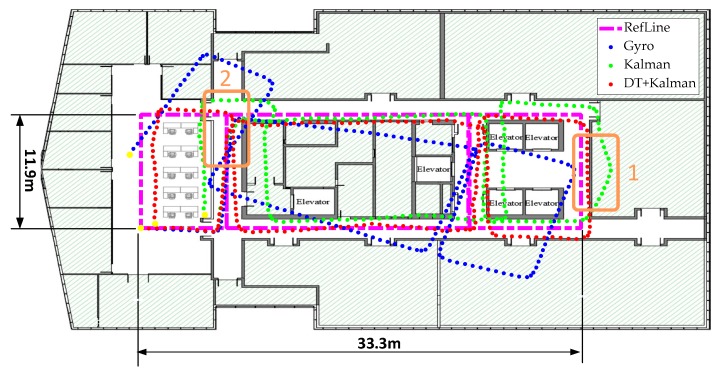
The result of the trajectory test by three different algorithms for indoor pedestrian inertial navigation. The walking time is 131.2 s indoors.

Figure 14 and Figure 15 show the heading, heading error, and positioning of the three methods compared to the actual routes (in magenta dots). The elevators at the position of orange frame 1 and the reinforced concrete column at orange frame 2 (Figure 15) heavily interfered with the geomagnetic field and caused deviations in the calculations of the heading angle. Using the Kalman filter only as in Figure 14a, the headings in orange frame 1 and orange frame 2 deviate noticeably from the baseline heading. Compared to the Gyro and Kalman methods, DT+Kalman has a significant effect in reducing the heading angle errors shown in Figure 14b. Using the proposed method can effectively solve irregular and short-term magnetic anomalies to achieve satisfactory performance for trajectory estimates.

The performance of the DT+Kalman algorithm is demonstrated quantitatively by the end point errors reported in Table 4. The proposed method improves the pedestrian inertial positioning accuracy by 74.6% in an indoor environment.

**Figure 16:**
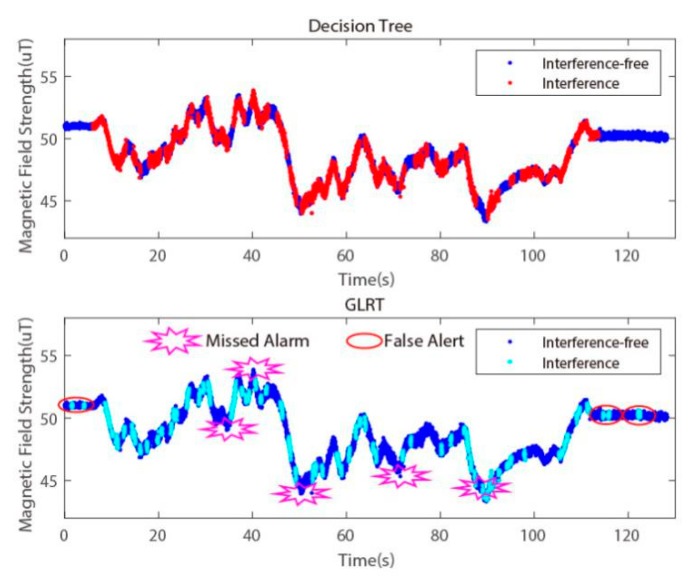
The magnetic data classification result generated by the Decision Tree and GLRT. (**a**) The red dots mark the interference measurement. (**b**) The cyan dots mark the interference measurement; the missed alarms and false alerts of the magnetic interference dots are also marked.

In order to prove that our algorithm can function in different indoor environments and to verify the performance advantages of the decision tree classification method, we also performed trajectory tests in the new environment and further compared it with the magnetic data classification method based on the GLRT from [20]. The classification effects of the decision tree and GLRT are exhibited in Figure 16. Compared with the decision tree, GLRT offers single threshold detection via the manual calculated method, which yields more false alarms and missed alarms when it filters the measurement magnetic data and will thus produce more errors in pedestrian navigation.

**Figure 17:**
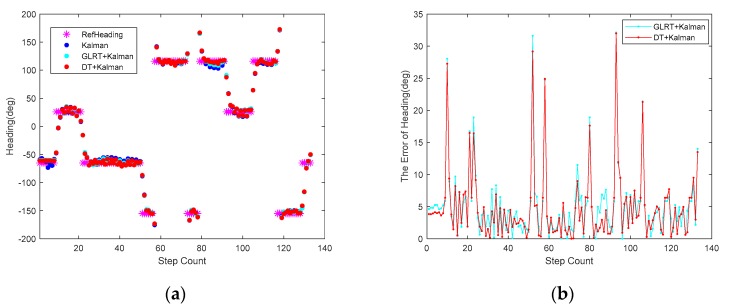
(**a**) Heading calculation of the GLRT+Kalman and DT+Kalman algorithms for indoor pedestrian inertial navigation. (**b**) The error of the heading angle for these two algorithms.

**Figure 18:**
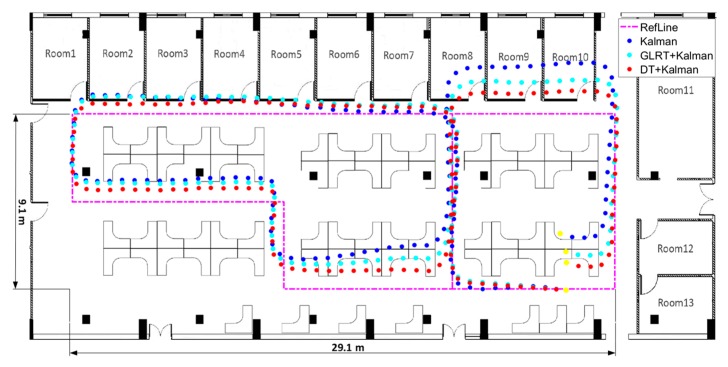
The result of the trajectory test based on the three algorithms in indoor pedestrian inertial navigation. The walking time is 128.3 s indoors.

For a more intuitive comparison, we tested both methods separately via a pedestrian inertial navigation experiment. Thanks to the more accurate interference magnetic data screening, the heading angle obtained by DT+Kalman is closer to the reference value in Figure 17a, and the error of the heading is smaller than that in Figure 17b. Meanwhile, higher positioning accuracy based on the proposed algorithm can be obtained, as seen in Figure 18.

The positioning errors of the pedestrian inertial navigation for the different methods are shown in Table 5. The result shows that the decision tree algorithm for classification based on multiple eigenvalues has a higher accuracy in magnetic data anomaly detection than the single-threshold GLRT method.

## 5. Conclusions

As the artificial magnetic perturbations in indoor affect the accuracy of the heading angle in pedestrian navigation, a magnetic anomaly processing method based on a decision tree was proposed. The undisturbed magnetic data are extracted from raw data and used to correct the attitude angle during the update phase of Kalman filtering. The proposed algorithm leverages the long-term stability of geomagnetic data in heading angle calculations and addresses the problem of irregular and short-term perturbation, thus increasing the accuracy of heading angle estimation and overall pedestrian navigation performance. Compared to the Kalman filter method, we found that the proposed algorithm effectively removed the non-Gaussian magnetic data and delivered higher accuracy, and the pedestrian positioning error was reduced from 4.61 m to 1.17 m by adopting the DT+Kalman method in the indoor navigation.

## Figures and Tables

**Table 1:** The magnetic data sequence with different feature values and categories.

No.	Fluctuation	Consistency	Gauss(Y/N)
**1**	a1	b1	Y
**2**	a2	b2	N
**3**	a3	b3	Y
**4**	a4	b4	N
⋮	⋮	⋮	⋮
**n**	an	bn	N

**Table 2:** The performance parameters of the VT-IMUs.

Sensors	Gyroscope	Accelerometer	Magnetometer
**Standard full range**	500 deg/s	8 g	8 Gauss
**Sensitivity**	65.6 LSB/deg/s	1024 LSB/g	4.35 milligauss
**Noise Density**	0.007 deg/s/√Hz (@10 Hz)	120 ug/√Hz	-
**Noise Floor**	0.07 deg/s (@200 Hz)	-	2 milligauss
**Non-linearity**	0.1% FS	0.5% FS	0.1% FS
**Cross-Axis Sensitivity**	2%	1%	0.2% FS/Gauss

**Table 3:** Comparison of the end point positioning error in outdoor track^1^.

Test Method	Gyro	Kalman	DT+Kalman
End point error	201.57 m	6.41 m	3.04 m

^1^ Note: The track is 219 m long and 131 m wide with a total length of 700 m.

**Table 4:** Comparison of the end point positioning errors in indoor track^2^.

Test Method	Gyro	Kalman	DT+Kalman
End point error	7.53 m	4.61 m	1.17 m

^2^ Note: The track is 33.3 m long and 11.9 m wide, with a total length of 90.4 m.

**Table 5:** Comparison of end point positioning errors in Indoor track^3^.

Test Method	Kalman	GLRT+Kalman	DT+Kalman
End point error	2.78 m	1.88 m	1.32 m

^3^ Note: The track is 29.1 m long and 9.1 m wide, with a total length of 94.6 m.
